# The Correction of Involutional Entropion of Eyelid by Lateral Strip Procedure

**DOI:** 10.4103/2006-8808.73616

**Published:** 2010

**Authors:** Kannan Balaji, Vijayalakshmi Balaji, Govindarajan Kummararaj

**Affiliations:** *Dr A Govindarajan Eye Hospital and Research Institute, Tiruchirapalli, Tamil Nadu - 620 017, India*

**Keywords:** Everting sutures, involutional entropion, lateral canthotomy, lateral strip procedure, tarsal plate

## Abstract

**Aim:**

To determine cosmetic and functional outcome following lateral strip procedure (LSP) for involutional entropion.

**Materials and Methods:**

This study was a prospective analysis of 15 patients (20 eyelids) of involutional entropion, who needed surgical repair. After thorough evaluation, the surgical treatment (LSP) was done in all 15 patients.

**Results:**

Cosmetic and functional outcome was excellent in all cases following LSP. No complications and recurrence were encountered in any case.

**Conclusions:**

LSP is simple, physiologic, easy and quick to perform as OPD procedure for involutional entropion under local anesthesia without hospitalization by a general ophthalmologist.

## INTRODUCTION

Involutional entropion is the most common type of entropion and usually involves the lower lids bilaterally, though not necessarily symmetrically.[1] The pathophysiologic mechanisms for the development of involutional entropion includes, horizontal eyelid laxity, detachment of retractors, overriding of the preseptal orbicularis, and enophthalmos.[2] Horizontal lid shortening has been shown to be the most important component in the surgical correction.[3] We believe that a simple surgical correction, which addresses all of the underlying anatomic abnormalities, yields the most acceptable results through lateral strip procedure (LSP).

## MATERIALS AND METHODS

This study was conducted at Dr A Govindarajan Eye Hospital and Research Institute, Trichy, Tamil Nadu, from April 2008 to September 2008. About 15 patients (5 bilateral cases) presenting to the OPD as well as through camp referral, with involutional entropion, especially needing the surgical repair, were taken up for the study after obtaining informed consent.

### Patient evaluation

Detailed history regarding the age and mode of onset of entropion was obtained. A complete ophthalmic examination was performed on all patients. This includes assessment of visual acuity, motility and pupillary responses, external evaluation and slit lamp biomicroscopy.

### External evaluation

The head posture of the patients, any facial asymmetry, palpebral fissure appearance and height, movement of the extra ocular muscles were noted. The contour of the eyelid and any evidence of pigmentation over the eyelids were noted. The position of the punctum, whether it was drawn away from the globe or apposed to the globe, was noted. The amount of horizontal lid laxity was also noted. The lower lid retractors’ weakening was also tested by the snap back test and distraction test. The other ancillary tests were to test for orbicularis muscle action, Bells phenomenon, Schirmer’s test, corneal sensation and staining to rule out dry eye and corneal surface irregularities due to entropion were performed in all the patients. The patients underwent the basic investigations such as complete blood count, random blood sugar, complete urinalysis, bleeding time, clotting time, BP check up. The surgical treatment (LSP) was planned for involutional entropion after physician’s clearance for medico legal purpose.

### Lateral strip procedure

#### Anesthesia

All the adults were operated under local anesthesia (2% lignocaine) with adrenaline. All of them were treated as OP patients.

The LSP involves shortening of the eyelid at the lateral canthal end. This corrects the anatomic defect. The canthal malpositions and shortening may be corrected simultaneously. The canthal angle shape is not altered. The procedure is easy and quick to perform. It is also useful in the management of anophthalmic socket and fitting of ocular prosthesis[4]. Tarsal plate fracturing was done for involutional entropion of upper lid.

### Basic steps

**Figure 1:**
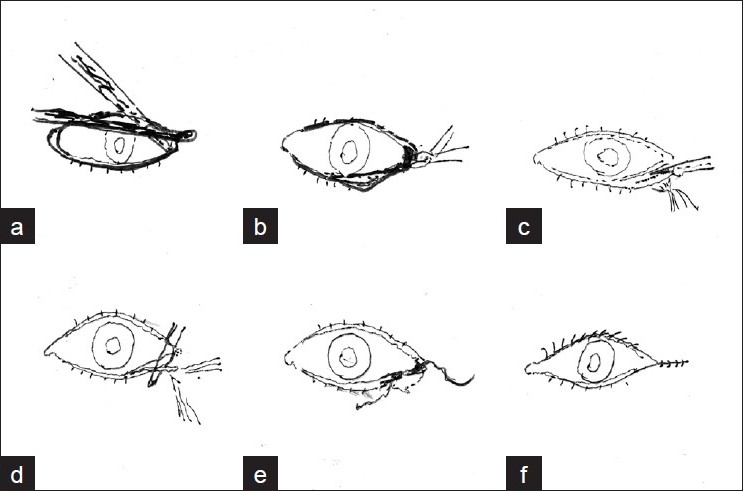
Basic steps. (a) Lateral cantholysis; (b) lateral canthotomy; (c) separation of gray line; (d) trimming of tarsal plate; (e) suturing of tarsal strip to periosteum and (f) canthal restructuring

**Figure 2:**
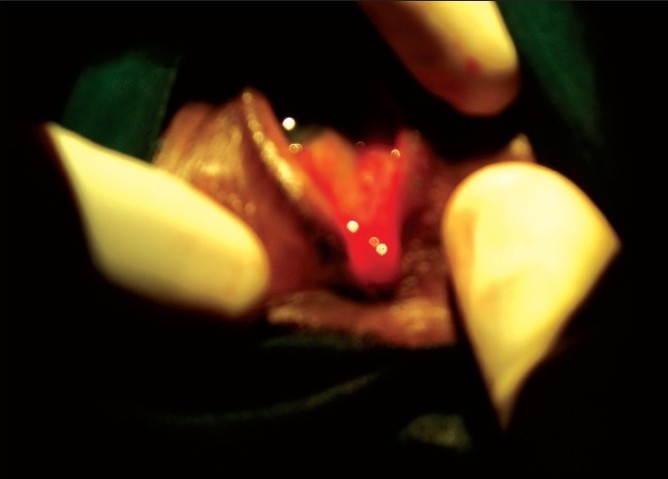
Lateral canthotomy and cantholysis

Lateral canthotomy was done till the lateral orbital rim was exposed with scissors. Lateral cantholysis was also done. Then, the eyelid was separated along the grey line with help of BP knife (no.15). An inferior cut was made horizontal to the above incision with knife. Excess conjunctiva was removed using conjunctival scissors. After assessing the redundancy, excess tissue was removed and the tarsal strip was sutured to the periosteum using 6/0 vicryl. The lash bearing anterior lamella was excised and skin closed with interrupted silk suture [Figure 1 (a-f) and Figure 2].

### Postoperative management

Tab. Ciprofloxacin 500 mg bd, Tab. Diclofenac sodium (50 mg) with Paracetamol (500 mg) bd, Tab. Ranitidine (150 mg) bd and multivitamins were given for 5 days. Antibiotic ointment was applied. Patients were examined for the first 2 days. Skin sutures were removed on the fifth day. The patient was reviewed every week for 6 weeks, at 3 months, 6 months and during last visit. During every visit, the patients were assessed for cosmetic and functional improvement.

### Main outcome measures

**Figure 3:**
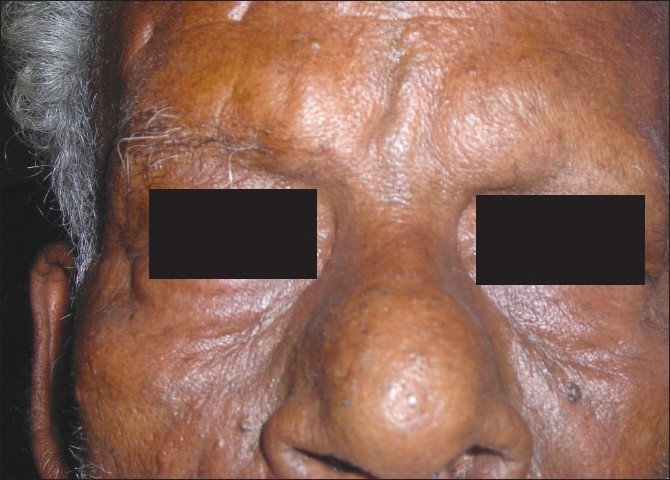
Preoperative photo showing involutional entropion in both eyes

**Figure 4:**
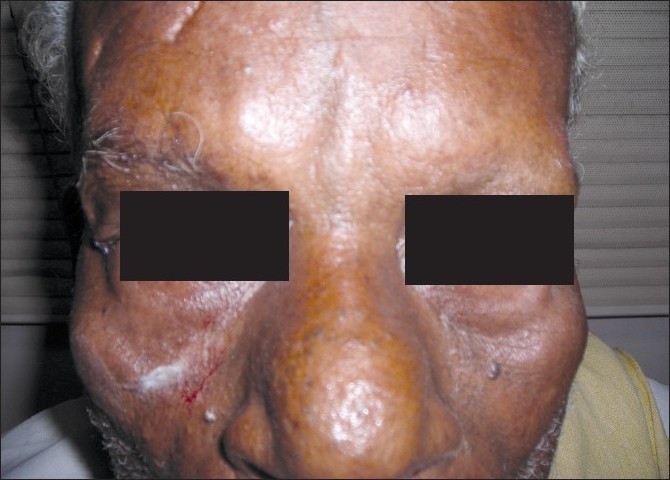
Postoperative photo showing normal eyelid position at rest in right eye compared to left eye

Cosmetic improvement should be defined as normal eyelid position at rest [Figures 3 and 4]. And functional improvement as inability to induce entropion on tetracaine provocation testing at or before the last visit.

## RESULTS

The mean age of the patients was 67.5 years (range 61–78 years). There were 12 males (80%) and 3 females (20%). The cases were followed up postoperatively for a mean duration of 13 months (range 3–20 months). All lids resolved spontaneously with good lid–globe apposition. LSP resulted in resolution of involutional entropion with 100% success rate.

No patient had any chance of anatomic recurrence during follow-up.

Neither intraoperative nor postoperative complications were encountered in any of the cases.

## DISCUSSION

LSP is the most commonly performed surgery for involutional entropion along with everting sutures; it can be effectively performed even by residents, fellows and attending supervising (oculoplastic) surgeon.[5] LSP is effective and safe with least recurrence and complication rate. It does not need any sophisticated instruments and is also so easy to comprehend and perform that it can be done by a general ophthalmologist anywhere. The complications of wound healing are rare due to extreme vascularity of the eyelids.[6] Cosmetic and functional outcome were excellent in all the cases of our study following surgical repair, with good long-term success rate.
